# 

**Published:** 1845-06-01

**Authors:** 


### BUFFALO MEDICAL JOURNAL,

EDITED BY

AUSTIN FLINT, M. D.

**Vol. 1.** **BUFFALO, JUNE, 1845.** **No 1.**

### CONTENTS:

Introductory,.......................................... ..........P. 1.

ORIGINAL COMMUNICATIONS.

Notes of an European Tour, by F. H. HAMILTON, M. D. Professor of Surgery in Geneva Medical College,.......................................... 3

Cases of Acute Rheumatism treated with Nitras Potassæ in large doses. By ALDEN S. SPRAGUE, M. D.......................................... 8

Case of Aortitis, with Autopsy, and Remarks. By GEO. N. BURWELL, M. D. 9

Case of Hydrophobia.......................................... 14

Cases of Midwifery, with twins, at different stages of development. By H. N. LOOMIS, M. D.......................................... 15

EDITORIAL, MEDICAL INTELLIGENCE, &c.

Reduction of dislocation of large Joints, by power derived from twisted rope... 16

Dose of the Extract of Conium.......................................... 16

Removal of seventeen inches of the small intestines, and recovery of the patient.......................................... 16

Transcendental Anatomy and Physiology.......................................... 17

Cyclopædia of Practical Medicine.......................................... 18

Copland's Medical Dictionary.......................................... 18

Marine Hospital at Cleveland.......................................... 18

New-York Journal of Medicine.......................................... 19

Oxalic Acid in Rhubarb or Pie Plant.......................................... 19

DR. HAMILTON's Lecture on Phrenology.......................................... 19

To Readers and Correspondents.......................................... 20

Meteorological Register.......................................... 21

BUFFALO:

C. F. S. THOMAS, PRINTER AND PUBLISHER,

Exchange Buildings, Third Story, Main-Street.

